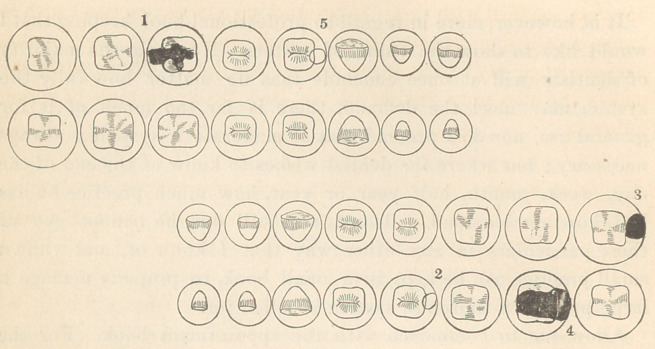# System as Applied to Instruments and Books

**Published:** 1884-02

**Authors:** W. St. George Elliott

**Affiliations:** London


### SYSTEM AS APPLIED TO INSTRUMENTS AND BOOKS.

BY DR. W. ST. GEO. ELLIOTT, LONDON.

Order is Heaven's first law—a law, alas, generally unappreciated by the majority of our profession, generally neglected by all.

Nature cannot work out her daily problems without that nice adjustment of means to ends, that law of order which renders easy what would otherwise be impossible; when we violate the law we entail upon ourselves additional labor and vexation of spirit, and fail where we should succeed. Enter the laboratory of a dentist of the present day, and see what indescribable confusion exists on every hand.

On a side shelf plaster models mixed up with some lumps of old wax, a broken tool or two, and some tobacco. Open a drawer and behold confusion worse confounded. Some odd sizes of screws, impression trays, old artificial sets, human teeth, dirty rags, and perhaps a stale piece of bread. Do not look behind the door, or under the table. *They* are not expected to be in order. Walk up stairs to the office: A Wilkerson chair perhaps meets the eye, but what neglect; the nickel plating has been allowed to assume that zinc-like color we are all so familiar with; the engine, too, has not been cleaned for months. A man who takes a pride in his operations should take some pride in his instruments, and if he has no assistant should keep his own in order, first seeing that they are in perfect working condition, and then that they are clean.

All instruments should be handsome as well as good ; attractive to a professional eye as well as efficient; with this end in view I have adopted the following plan, which has answered perfectly. All ordinary instruments are made with socket handles, of which I have in use nearly a hundred. These all have celluloid centers in different colors, and are made as follows: A piece of celluloid the necessary size, say two inches long and one-half inch thick, is drilled through lengthwise. Together with the steel handle it is heated over a Bunsen burner, and when the celluloid is soft the steel is driven through it as far as may be required, brass ends added and soft soldered on. It is then put on the lathe and the celluloid and end pieces turned down to suit, and nickel plated. This forms a permanent handle which for beauty and efficiency leaves nothing to be desired. The points are changeable in the sense that they are replaced with new ones when worn out. The system I have adopted for excavators is this: All are in one drawer in a row from right to left, commencing with the right angled hoes, ten each, in dark red; the oblique hoes, ten, light red ; the right angled hatchets, dark blue, and the oblique angled, light blue; large points always to the right, graduated to the left, so that the hand is instantly placed upon the instrument needed, and when mixed with others on the table they can be singled out at once by color, etc. The pluggers cannot be systematized so thoroughly ; those of a kind have handles nearly alike, the difference being in the shape ; *e. g.* Varney's set is in mottled celluloid, each handle different in design.

It is, however, more in regard to professional book-keeping that I would like to show the value of system. No doubt the majority of dentists will at once conclude that no matter how fully the system may meet the demand, there is far too much of it for general use, nor do I pretend that so elaborate a system is always necessary; but where the dentist wishes to know at the end of the day, week, month, half year or year, how much practice he has had, how much earned, collected, and still due, he cannot get all these particulars in any other way that I know of, and while a small practice can be kept in a small book, to properly manage a large practice a thorough system is called for.

Allow me to commence with the appointment book. For the purpose of reference I label this book with the year, and commence it on January 1st. All future appointments are made in pencil, as changes are inevitable, but as soon as the day is over they are put in ink, with the time given to each. Should any one fail to keep an appointment, it is so marked. This book is kept as a permanent record of appointments for the year, and I have often found it of value. Next in order we have the Ledger, a reduced copy of which appears on next page.

This is the third form I have used. It will be seen that the median line divides the right from the left, the temporary teeth being under the permanent ones.* Of course the teeth may be arranged in any way; the main object in adopting the one illustrated is that the space occupied is less. You will notice several new features in these records. *First.* The A A, approximate age, a matter of value if the testimony of your records is to be complete. *Second.* We have the time occupied at each sitting, then the usual columns for debtor and creditor; a column P D, or probable duration of work, which is estimated by the character of the case, cleanliness of the patient, etc.; generally putting it at ten years for a good average case in gold, and when temporary work, as oxy-phosphate, etc, is used, the durability is generally put at one year; in the case of amalgam the same time is given as for gold. Then we have what is to me the feature of greatest value—the place for remarks,

The cuts of the temporary teeth are necessarily omitted.—Ed.

Hon'ble Miss Smith,
Sent by Dr. Smith, of Melbourne.
Pine Shanty, Undercloud, Middlesex.

| Date, 1882. | | No. | Operation. | Time. | Dr. £ | Cr. £ | P.D.[1] | AA[2] 99 Remarks. |
|---|---|---|---|---|---|---|---|---|
| July, | 25 | 1 | Gold Filling................ | | 10.10 | | 10 | Tin foil at cervicle edge. Electric Mallet. Sensitive. |
| " | " | 2 | Poulson's Cement. ....... | 1.55 | 1. 1 | 11.11 | 1 | Soft chalkey tooth. Nerve nearly exposed. |
| Aug. | 6 | 3 | Amal. Fill. (Nickold's)..... | .30 | 1. 1 | | 1 | Patient irritable. Visit Aug. 9th. 0. |
| " | 10 | 4 | Gold Filling...... .. .... | 3. | 15.15 | | 10 | Nerve Canals packed with carbolized paper. Platinum pin in distal root. |
| " | 11 | 5 | Gutta Percha Fill. (Oliver's) | .20 | 1. 1 | | 1 | Temporary filling to push away gum. |
| | | | | | 17.17 | | | Bill Aug. 15th. Visit Aug. 12th, 0. do. Aug. 13th, 0. |
| " | 16 | | | | | 17.17 | | |

1. P. D.—Probable duration. 2. AA.—Approximate age. Gold Fills. are located in red. Amal. Fills. are located in black. White Plastics are located by a circle.

a line of about four inches for each operation. The character of these observations is shown on the page furnished. It also shows that the patient paid two visits for which no charge was made, that the work was finished August 11th, bill sent in on August 15th, and paid August 16th. The patient's first visit, as the record shows, was paid for at the time. Thus we have a recorded history to each operation, either pathological, as pericementitis, alveolar abscess, etc., etc., or operative, as the use of tin foil mixed with gold, the character of the surface, whether made with Pack's pellets, folded foil, or heavy numbers. This enables us to study the comparative wearing qualities of the different foils. With many dentists this ledger would give all the information they desire to have. I am not, however, satisfied with this. In conducting a large practice I wish to know what is due each day, week, month, quarter, etc., and for this purpose when my secretary has posted the ledger for the day, she makes the necessary entries in the Day Book, a sample of which follows:

DAY BOOK.

July, 1882.

| Date. | NAME. | Opera. | Dr. 1 | Cr. on Dr. 2 | Cr. 3 | Bal. 4 |
|---|---|---|---|---|---|---|
| 3 | Hunter, Miss | | | | 1.1 | |
| " | Hunter, Mrs | 2 Gold | 8.8 | | | 8.8 |
| " | Payne, Miss | 1 Gut. P. Clean. | 2.2 | | | 2.2 |
| " | Mercer, Col | 1 Gold. | 1.1 | 1.1 | | |
| " | Smith, Mrs | Treat. 1 Gold. | 3.3 | | | 3.3 |
| " | McPherson, Mr | 1 Gold. Treat. 1 Amal. | 5.5 | | | 5.5 |
| " | Gray, Mrs | 1 Gut. P. 1 Toss. | 3.3 | | | 3.3 |
| " | Gray, Miss | Reg. Plate. | 3.3 | | | 3.3 |
| 4 | Hunter, Mrs | | | | 8.8 | |
| " | Brown, Miss | | | | 16.16 | |
| " | Davies, Mr | | | | 26.5 | |
| " | Norton, Mr. H | Treat. 1 Amal. | 2.2 | | | 2.2 |
| " | Stearn, Miss | 2 Gold. | 9.9 | | | 9.9 |
| | For part of week (2 days) | | 37.16 | 1 1 | 52.10 | 36.15 |

1.—Debtor, 2.—Creditor on Debtor. 3.—Credit on old account. 4.—Balance due on day's work.

It shows the name of the patient, the operations performed for each, the price, and cash account. Another column for payments on back accounts, and another for the balance due. The page given shows that on the third of the month Miss Hunter paid a back account of one guinea. Mrs. Hunter had two gold fillings put in, charged eight guineas, and as they were not paid for at the time they appear also in the column of balance due. Colonel Mercer's fee appears in the second, or creditor on debtor column, because he paid it at the time. This book is handed to me after posting, and I verify it. I can thus turn to any day in the year and see what was done. The monthly and quarterly statements are but compilations from the day book, at the end of which there is a schedule of earnings, month by month, and year by year.

### MONTHLY REPORT.

### AUGUST.

| | £ | s. | d. |
|---|---|---|---|
| Gross income of Office | 545 | 19 | |
| Total cash receipts | 415 | 10 | |
| Total balance due | 1770 | 4 | 6 |

| Expenditure | £ | s. | d. |
|---|---|---|---|
| Mechanical Assistant | 13 | 10 | |
| Office Secretary | 10 | | |
| " Boy | 3 | | |
| Petty Office expenses | 10 | 6 | 3½ |
| Total office expenditure | 36 | 16 | 3½ |

### SEMI-ANNUAL REPORT.

### JAN. TO JULY.

| | £ | s. | d. |
|---|---|---|---|
| Gross income of Office | 2269 | 0 | 0 |
| Total cash receipts | 1917 | 6 | 9 |
| Total balance due | 1639 | 15 | 6 |
| Office Expenditures | 171 | 12 | 1 |

### INCOME BY MONTH, IN GUINEAS.

| | 1880 | 1881 | 1882 | 1883 | 1884 |
|---|---|---|---|---|---|
| January | 120 | 215 | 225 | | |
| February | 300 | 300 | 329 | | |
| March | 350 | 375 | 402 | | |
| April | 310 | 341 | 376 | | |
| May | 250 | 425 | 465 | | |
| June | 370 | 450 | 469 | | |
| July | 321 | 501 | 545 | | |
| August | 40 | 50 | 69 | | |
| September | 350 | 357 | 360 | | |
| October | 270 | 275 | 280 | | |
| November | 390 | 400 | 410 | | |
| December | 400 | 421 | 425 | | |

Finally, there is a general index, where the daily accounts go through three ledgers, or more, A, B, C, etc. The general index shows in what book or books the name appears.

Outside of purely professional books I keep an office expense book, in which are entered all office expenditures, so that I can tell at the end of each month what my dental depot account is, how much I pay for mechanical work, how much for sundries, as files, pumice-stone, etc. Once a year I have a public accountant go over all the books, and correct any mistakes that may have occurred. In the laboratory I try to keep as thorough a system with all my tools, so that time is saved by having just what is wanted where I can place my hand upon it, and I try to avoid lumbering up the table with tools of all kinds, helter-skelter.

Although so much satisfaction is derived from system, yet go where you will, it is the absence of it that you notice. Who ever saw a watchmaker or jeweler keep his tools in order, and yet while admitting that time is money, and that system saves time, they always act as if it might be good for others, but not for them. Finally, let me implore you to cultivate that of which nature has given us so many examples, system. We all wish to excel in our chosen calling; then let us take the means at our disposal for our assistance, systematize our instruments, our books, our ideas, our operations.

## Figures and Tables

**Figure f1:**
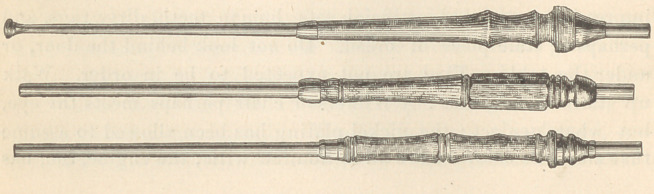


**Figure f2:**